# Reducing Competition of Pepsin in Aflatoxin Adsorption by Modifying a Smectite with Organic Nutrients

**DOI:** 10.3390/toxins12010028

**Published:** 2020-01-02

**Authors:** Ana Luisa Barrientos Velazquez, Youjun Deng

**Affiliations:** Department of Soil and Crop Sciences, Texas AM University, College Station, TX 77845, USA; yjd@tamu.edu

**Keywords:** aflatoxin, adsorption, nutrient-montmorillonite

## Abstract

Carcinogenic aflatoxins can be inactivated by smectites (e.g., montmorillonite) through adsorption and degradation. Proteins in gastric fluids can reduce smectite’s adsorption capacity for aflatoxins. The objective of this study was to evaluate the efficiency of smectites modified with organic nutrients in restricting the influence of proteins on aflatoxin adsorption. Arginine, histidine, choline, lysine, and vitamin B1 were selected to occupy part of the interlayer space of montmorillonite to achieve a smectite structure more selective for aflatoxin adsorption, but not for the large protein molecules. The unmodified montmorillonite had a maximum adsorption capacity of 0.2 mol/kg in the presence of pepsin. The vitamin B1-montmorillonite showed significant improvements in the aflatoxin affinity constant from 0.065 to 0.201 μM${}^{-1}$ and the aflatoxin adsorption to 0.56 mol/kg. Choline-montmorillonite and histidine-montmorillonite showed a moderate increase in AfB1 adsorption. Arginine-montmorillonite and lysine-montmorillonite showed a slight increase in the adsorption capacity, but did not improve the affinity constant. The XRD results indicated that pepsin could still access the interlayer of nutrient-montmorillonite complexes. The intercalation of organic nutrients into the interlayer space of montmorillonite improved the AfB1 adsorption by restricting the adsorption of pepsin.

## 1. Introduction

Aflatoxins are carcinogenic secondary metabolites produced by fungi *Aspergillus*. Numerous adsorption experiments have demonstrated that smectites have a high adsorption capacity for aflatoxin. Montmorillonites can adsorb up to 20% (by mass) of aflatoxin. Animal experiments have shown that smectites can effectively reduce aflatoxin toxicity by adsorbing aflatoxins in the gastrointestinal tract when incorporated into an aflatoxin contaminated feed [1,2,3].

Variable temperature X-ray diffraction analysis demonstrated that aflatoxin molecules were adsorbed in the interlayer space of smectites and occupied the hydrophobic sites [4]. It was also observed that the valence and size of the exchangeable cation influenced the adsorption capacity and affinity for aflatoxin molecules: the divalent cations had stronger ion–dipole interaction with the carbonyl groups on the AfB1 molecule than the monovalent cations. Furthermore, cations with smaller hydration radii induced greater adsorption because less water was brought into the interlayer. Based on the effect of the exchangeable cation on aflatoxin adsorption and the infrared responses, the proposed adsorption mechanism was an ion–dipole interaction between the carbonyl group of the AfB1 molecule and the exchangeable cation under low humidity conditions and H-bonding between the hydrated cation and the C = O groups [4]. Further experiments also demonstrated that the smectites’ layer charge affected the aflatoxin adsorption, and a cation exchange capacity (CEC) of 85 cmol${}_{c}$/kg was optimal to achieve the highest aflatoxin adsorption [5]. The adsorption capacity of high charge smectites was improved when the CEC was reduced from 120 cmol${}_{c}$/kg to 85 cmol${}_{c}$/kg. An optimum layer charge in combination with specific cation saturation appeared to be a combination that would result in an enhanced aflatoxin adsorption.

In previous experiments, the efficiency of a bentonite (4TX from Gonzales, TX, USA) with a high aflatoxin adsorption capacity was tested in an in vivo experiment [6]. The batch adsorption isotherms of the 4TX bentonite had an aflatoxin adsorption capacity of 0.45 mol/kg. When this bentonite sample was tested in an animal trial, the chickens fed an aflatoxin contaminated diet with clay showed 21% improvement in body weight in comparison to the aflatoxin diet with no clay. However, the body weight of the chickens in the AfB1 contaminated feed amended with clay (average body weight of 371 g) was significantly lower than the non-aflatoxin control group (average body weight of 678 g).

Despite numerous successes in adsorption experiments that have demonstrated the high capacity of the clays in adsorbing aflatoxin in water, a complete animal recovery from aflatoxicosis in animal trials has not been observed after adding smectites to aflatoxin contaminated feed. Moreover, the quantity of incorporated smectite in the feed trials should adsorb several orders of magnitudes or more aflatoxins than those in the contaminated feed. The lower than expected performance of smectites in animal trials can be at least partially attributed to the complex compositions of the gastrointestinal fluids.

Comparing the adsorption capacities of two smectites in water and in corn meal solution showed a significant AfB1 adsorption reduction in the corn meal solution [7]. The low aflatoxin adsorption in corn meal was attributed to the competition of soluble compounds from corn meal, yet no details about these competing compounds were given.

A recent study indicated that the Ca-montmorillonite’s adsorption capacity (Qmax) for aflatoxin reduced from 0.52 mol/kg in water to 0.32 mol/kg in simulated gastric fluid [8]. Divalent cation Ca and Ba saturated smectite had greater adsorption capacity than Na-smectite in water, yet the difference among the cation saturated montmorillonite diminished in simulated gastric solution. The poor aflatoxin adsorption in simulated gastric fluid was attributed to the intercalation of proteins such as pepsin into the interlayer of smectite by cation exchange. Large protein molecules altered the interlayer environment and occupied the adsorption sites for aflatoxins.

To retain the high aflatoxin adsorption capacity of smectites in gastric fluids, the accessibility of proteins to the interlayer of smectite must be blocked by certain treatments, yet the treatments should keep the adsorption sites available for aflatoxin molecules.

A common approach to modify the interlayer of smectites involves the intercalation of a cationic surfactant or small organic cations to increase the surface hydrophobicity of the clay. Jaynes et al. [7] intercalated a montmorillonite with choline, carnitine, phenyltrimethylammonium (PTMA), and hexadecyltrimethylammonium (HDTMA) and investigated the aflatoxin adsorption capacity of the modified montmorillonite in a corn flour solution. The presence of carnitine and choline increased the adsorption of aflatoxin in comparison to the non-modified montmorillonite. The most common organic cationic surfactants such as HDTMA can raise a concern about their toxicity when incorporated in feed. In a recent HDTMA toxicological study, Revee et al. [9] tested the desorption of HDTMA from surfactant modified zeolite and observed that at the concentration released, the surfactant was toxic to some soil microflora. Abdel et al. [10] tested the aflatoxin and ochratoxin adsorption capacity of an organic montmorillonite modified with another cationic surfactant cetyltrimethylammonium bromide (CTAB) in rats. The animals under the mycotoxin contaminated diet amended with the organic-montmorillonite showed a recovery in biological indicators, demonstrating the effective mycotoxin adsorption by the modified montmorillonite. The toxicity of the CTAB-montmorillonite was also assessed, and the biological parameters were similar to the control group, which suggested that the modified montmorillonite was safe to animals. Although it did not appear that the CTAB-montmorillonite caused noticeable toxicity in the parameters tested, CTAB is a toxic compound to handle [11].

**Figure 1:**
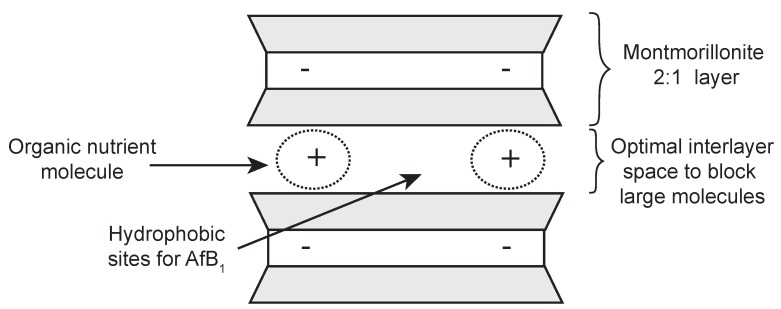
Schematic model of the interlayer modification of smectite with organic nutrients.

To avoid the possible toxicity of the modifying organic or inorganic compounds, it would be more desirable to use non-toxic or low toxicity compounds such as food ingredients or organic nutrients to achieve an optimal interlayer space and surface polarity for selective adsorption of AfB${}_{1}$ (Figure 1). Barrientos et al. [8] observed that when vitamin B1 was added into the simulated gastric solution, the AfB${}_{1}$ adsorption was greater in the vitamin-pepsin mix solution than in the pepsin only solution. This suggested that vitamin B1 might be able to restrict the access of pepsin into smectite.

The objective of this study was to evaluate the effectiveness of smectites’ modifications with five common organic nutrients in the aflatoxin B1 adsorption in simulated gastric fluid by restricting the interlayer access of proteins such as pepsin. Our goal was to let organic nutrients occupy part of the interlayer space of smectites to achieve a more desired interlayer space for aflatoxin adsorption, but not for the large protein molecules.

## 2. Results

### 2.1. Intercalation of Nutrients into Montmorillonite to Redefine the Interlayer Space and Polarity

**Figure 2:**
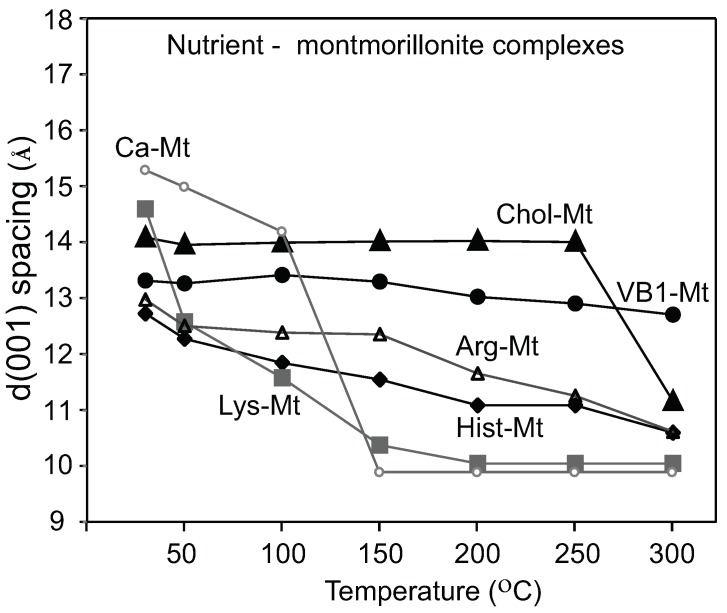
Interlayer heating stability of the unmodified montmorillonite and the five nutrient-montmorillonite (Mt) complexes.

The XRD diffraction patterns of the nutrient-montmorillonite (Mt) complexes showed a range of d(001)-values from 14.6 to 12.8 Å at room T (Figure 2). The intercalation of the nutrient molecules in the interlayer of montmorillonite was confirmed by the response of the d(001) spacing in the sequential heating from 50 to 200 ${}^{\circ }$C: the Ca-Mt collapsed to 10 Å below 150 ${}^{\circ }$C, but choline-montmorillonite (Chol-Mt) and vitamin B1-montmorillonite (VB1-Mt) complexes maintained an expanded interlayer spacing at 14 and 13 Å, respectively, up to 250 ${}^{\circ }$C. For arginine-montmorillonite (Arg-Mt) and histidine-montmorillonite (Hist-Mt) complexes, the d-values decreased from 13 Å to 11 Å at 250 ${}^{\circ }$C. The lysine-montmorillonite (Lys-Mt) complex showed a significant d-value decrease after heating at 100 ${}^{\circ }$C, and further heating showed a collapsed interlayer at 10 Å, which suggested that the montmorillonite was not completely saturated with lysine. Maintaining an expanded interlayer after heating at 200 ${}^{\circ }$C indicated the presence of the nutrients in the interlayer spaceof montmorillonite. The VB1-Mt complex maintained the expanded interlayer of 13 Å at 300 ${}^{\circ }$C, while the other nutrient-Mt complexes collapsed to below 11 Å at 300 ${}^{\circ }$C.

**Figure 3:**
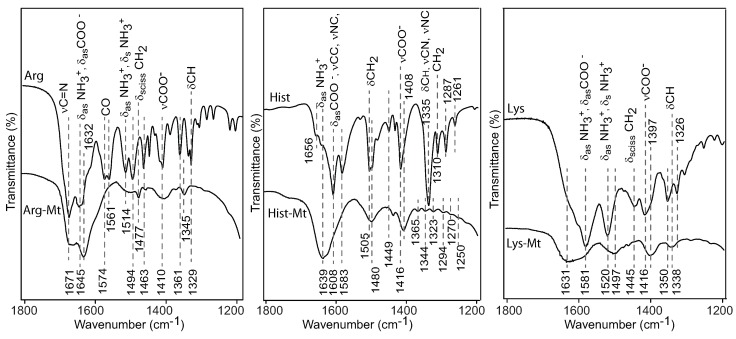
FTIR spectra of arginine, histidine and lysine modified montmorillonite and their corresponding organic solutions.

The intercalation of the nutrient compounds in the montmorillonite was confirmed with the FTIR spectra of the nutrient-Mt complexes. The C=N vibration at 1671 cm${}^{-1}$ in arginine shifted to 1632 cm${}^{-1}$ in the Arg-Mt complex (Figure 3). Interlayer water contributed to the broadening and intensity of the 1632 cm${}^{-1}$ band. The doublet at 1574 and 1561 cm${}^{-1}$, attributed to C=O [12], was observed in the free arginine spectrum, but disappeared in arginine-montmorillonite. Additional bands of NH${}_{3}^{+}$ and COO${}^{-}$ at 1514 and 1410 cm${}^{-1}$ became weaker in the arginine-montmorillonite sample. Similar band shifts were observed for Hist-Mt and Lys-Mt. Free histidine showed the protonated NH${}_{3}^{+}$ band at 1639 cm${}^{-1}$ and two strong bands mainly due to COO${}^{-}$ vibration, but the Hist-Mt complex showed only a broad band at 1639 cm${}^{-1}$. An additional COO${}^{-}$ vibration at 1416 cm${}^{-1}$ in histidine shifted to 1408 cm${}^{-1}$ in the Hist-Mt complex spectrum. The Lys-Mt complex showed weak bands possibly indicating low lysine saturation or the adsorbed lysine was removed during washing.

**Figure 4:**
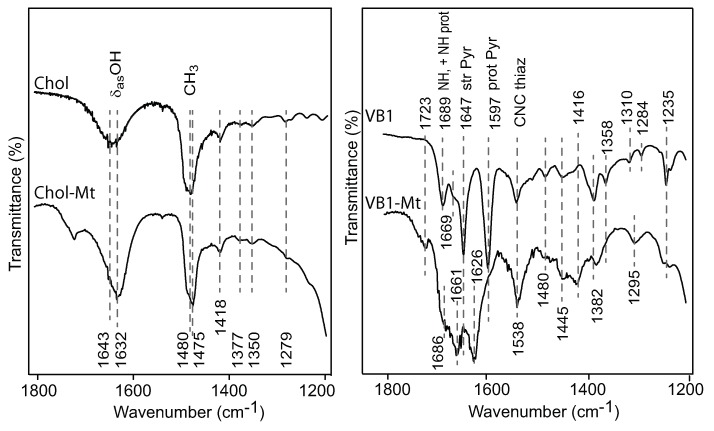
FTIR spectra of choline and VB1 modified montmorillonite and their corresponding organic solutions.

The 1643 cm${}^{-1}$ band in the choline spectrum, attributed the asymmetric O-H vibrations [13], shifted to 1632 cm${}^{-1}$ in the Chol-Mt complex. No other significant band shifts were observed in the Chol-Mt complex compared to free choline (Figure 4). Both choline and the Chol-Mt complex showed strong bands at 1480–1475 cm${}^{-1}$ due to -CH${}_{3}$ bending vibrations. Additional scissoring and bending vibration were observed at 1418 cm${}^{-1}$ and 1377 cm${}^{-1}$, respectively [14].

When vitamin B1 was adsorbed by montmorillonite, the 1689 cm${}^{-1}$ band in the VB1 spectrum due to the bending vibration of the protonated NH${}_{3}^{+}$ appeared as a weak shoulder in the VB1-Mt complex. The band at 1647 cm${}^{-1}$ in the thiamine spectra possible shifted to 1626 cm${}^{-1}$ in the nutrient-Mt complex. The strong band at 1596 cm${}^{-1}$ in the vitamin B1 became a weak shoulder in the VB1-Mt spectrum, indicating the absence of unprotonated species [15].

### 2.2. Reduced Intercalation of Protein in Nutrient-Montmorillonite Complexes

**Figure 5:**
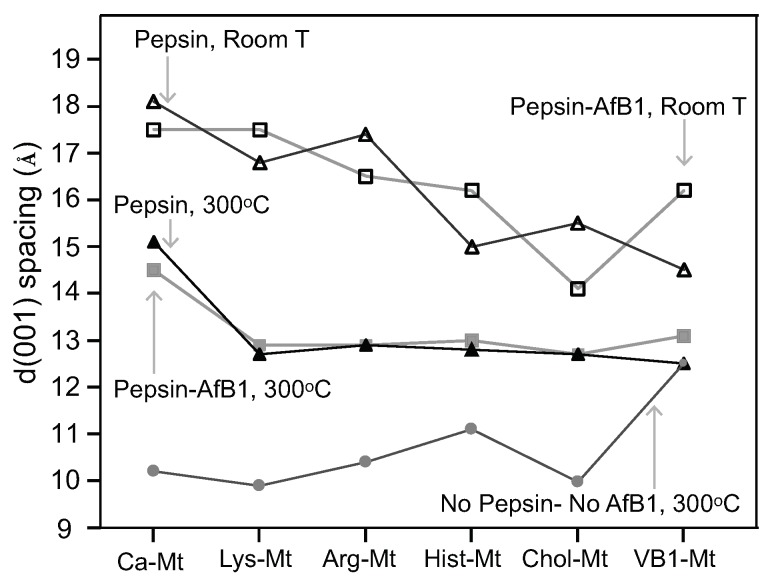
Interlayer 001 d-values of montmorillonite clay-organic complexes loaded with pepsin and pepsin + AfB${}_{1}$ at room T and after heating at 300 ${}^{\circ }$C.

The adsorption of pepsin resulted in different interlayer expansions in the montmorillonite (Figure 5). The basal spacing d(001) of the unmodified montmorillonite (Ca-4TX) expanded to 18 Å after pepsin adsorption at room T. Arg-Mt and Lys-Mt complexes were also expanded to 17 Å by pepsin, but Chol-Mt, Hist-Mt, and VB1-Mt complexes were expanded to 15 Å by pepsin. After heating to 300 ${}^{\circ }$C, the basal spacing of the pepsin-Ca-Mt complex collapsed to 15 Å, while all the nutrient-Mt complexes collapsed to 12–13 Å. The responses of basal spacings to heat suggested the adsorption of pepsin in the nutrient-Mt complexes persisted, but not to the extent as in Ca-Mt (Figure 2).

**Figure 6:**
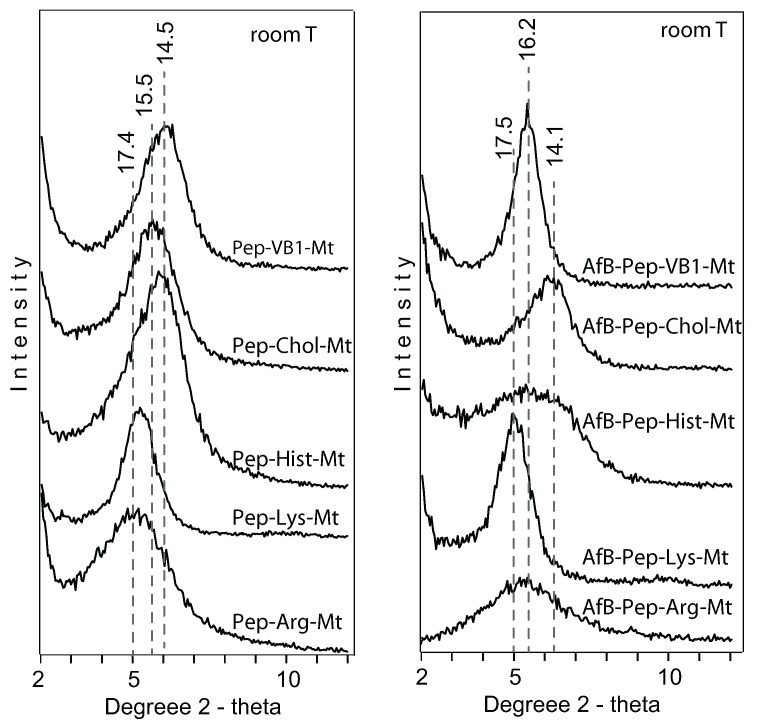
X-ray diffraction patterns of nutrient-montmorillonite complexes with pepsin and pepsin + AfB${}_{1}$ in the interlayer at room T.

When both AfB1 and pepsin were adsorbed, the Ca-Mt d001value was slightly lower than pepsin-Mt at room T (Figure 5). A similar trend was observed in Arg-Mt and Chol-Mt complexes, suggesting less protein was adsorbed when AfB1 was in solution. The opposite was observed in Lys-Mt, Hist-Mt, and VB1-Mt, where the adsorption of aflatoxin expanded the interlayer. In the Lys-Mt complex, the differences in the basal spacing between adsorbed aflatoxin and pepsin was attributed to moisture conditions because their XRD patterns were very similar. The AfB1-Pep-Hist-Mt complex showed a broad non-well defined peak in comparison to Pep-Hist-Mt (Figure 6). The AfB1-Pep-VB1-Mt complex expanded to 16.1 Å, which was attributed to the vitamin B1 effectively blocking protein from the interlayer, but allowing for AfB1 adsorption. Heating the AfB1-Pep-nutrient-Mt complex to 300 ${}^{\circ }$C collapsed the interlayer between 12 and 13 Å, similar to the nutrient-Mt with adsorbed pepsin only.

**Figure 7:**
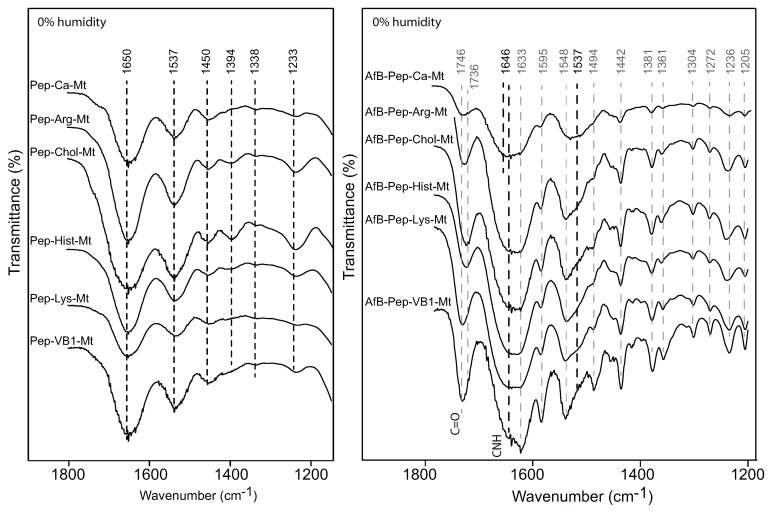
FTIR spectra of organic-montmorillonite complexes loaded with pepsin and pepsin + AfB${}_{1}$.

The FTIR spectra of the Pep-nutrient-Mt complexes confirmed the presence of pepsin in the complexes as the major pepsin bands observed in the Ca-Mt spectrum were also present in the nutrient-MT complexes (Figure 7). The FTIR spectra after the adsorption of aflatoxin by the nutrient-Mt complexes in the presence of pepsin showed strong bands of both aflatoxin and pepsin. The aflatoxin bands in the nutrient-Mt complexes were more intense than those in the Ca-Mt, which correlated with their higher AfB1 adsorption capacity more than the unmodified Ca-Mt. The presence of pepsin contributed to the broadening of the 1548 cm${}^{-1}$ band in Ca-Mt, but this band became sharper in the nutrient-Mt complexes. The 1537 cm${}^{-1}$ band occurred as a shoulder for all the nutrient-Mt complexes except for vitamin B1-Mt, in which this band was not present. The absence of the 1537 cm${}^{-1}$ shoulder and the weaker 1646 cm${}^{-1}$ band in the VB1-MT complex suggested that less pepsin was present in comparison to the other nutrient-Mt complexes. This observation corroborated the adsorption data that indicated increased preference of VB1-Mt for aflatoxin and reduced the access of pepsin.

### 2.3. Aflatoxin Adsorption of Nutrient-Montmorillonite Complexes in the Presence of Pepsin

**Figure 8:**
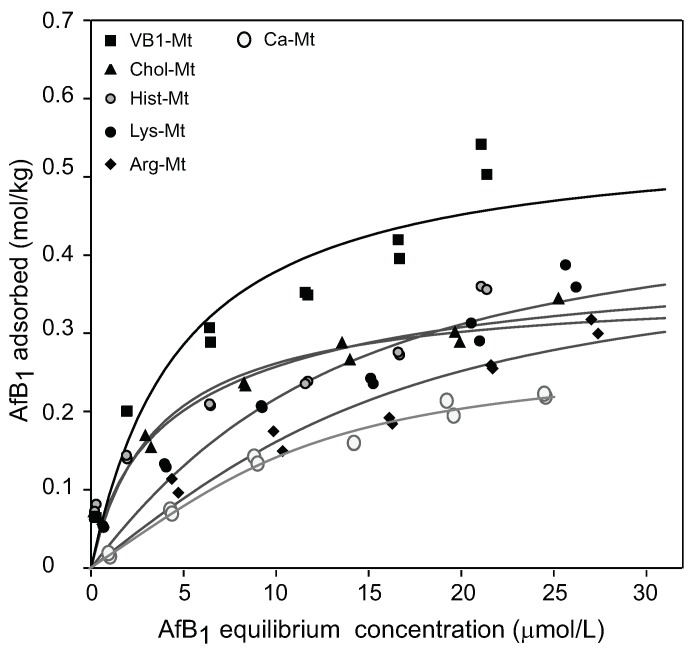
Aflatoxin adsorption isotherms of the nutrient-montmorillonite complexes in the presence of pepsin.

In the presence of pepsin, the nutrient-Mt complexes improved the aflatoxin adsorption as compared to Ca-Mt (Figure 8). The Arg-Mt and Lys-Mt complexes showed a slight increase in the maximum adsorption capacity (Qmax) of 0.42 and 0.48 mol/kg, respectively, but the affinity constants were still low (Table 1). Hist-montmorillonite had the lowest Qmax of 0.36 mol/kg, but the affinity constant for aflatoxin was greater than Lys-Mt and Arg-Mt. Chol-Mt showed a moderate adsorption (Qmax = 0.41 mol/kg) and improved the affinity constant. VB1-Mt had the highest adsorption capacity of 0.56 mol/kg and a significantly improved affinity constant (Figure 8).

### 2.4. Interlayer Accessibility of AfB1 Molecules to Nutrient-Montmorillonites

**Figure 9:**
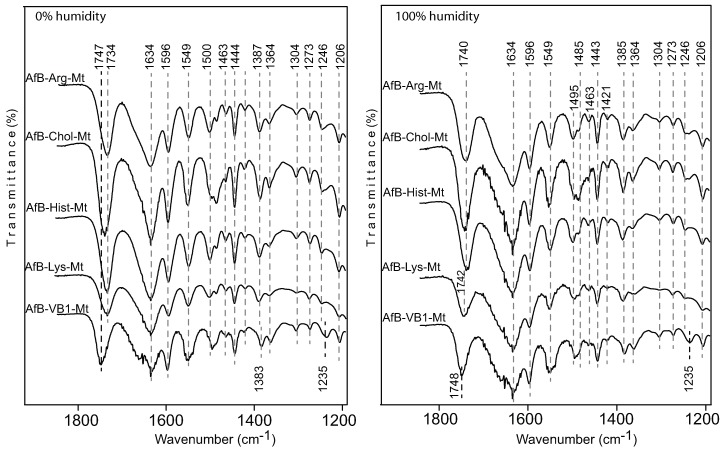
FTIR spectra of aflatoxin B1 adsorbed by the nutrient-montmorillonite at 0% and 100% humidity complexes.

The FTIR spectra of the Aflatoxin-nutrient-Mt complexes showed the major bands of adsorbed aflatoxin molecules in the region from 1800 to 1200 cm${}^{-1}$ (Figure 9), similar to the spectra of the AfB1-Pep-nutrient-Mt complexes. The strong bands at 1548 and 1494 cm${}^{-1}$ in the AfB-nutrient-Mt complex formed in water were also observed in the aflatoxin nutrient-Mt in the presence of pepsin, but these bands were absent in Ca-Mt, indicating more intercalated aflatoxin molecules in the nutrient-Mt complexes than Ca-Mt. Unlike the AfB1-Mt complexes, the FTIR bands of the AfB-nutrient-Mt complexes showed minimal response to humidity changes. At high humidity, the aflatoxin C=O band occurred at 1740 cm${}^{-1}$ in the Arg-Mt, Chol-Mt, Hits-Mt, and Lys-Mt complexes, while the AfB1-VB1-Mt complex showed the carbonyl band at 1748 cm${}^{-1}$. When the humidity was reduced, the arginine-, choline-, histidine-, and lysine-Mt complexes showed a shift from 1740 cm${}^{-1}$ to 1734 cm${}^{-1}$, but the C=O band was not affected by the humidity change in the AfB1-VB1-Mt complex. Another noticeable difference in the AfB1-VB1-Mt complex was the presence of the 1235 cm${}^{-1}$ band, which appeared to be a shift from the 1246 cm${}^{-1}$ band as observed in all other samples.

## 3. Discussion

### 3.1. Varied Degrees of Intercalation of Organic Nutrients in Montmorillonite

Montmorillonites have been widely used as adsorbents for numerous organic compounds such as amino acids, vitamins, and various medical molecules. Early studies demonstrated the adsorption and interaction of amino acids by smectites. Jang et al. [17] investigated the interaction between interlayer adsorbed lysine and the exchangeable cations. The lysine-montmorillonite IR band positions shifted in response to the transition metal present in the interlayer. The authors concluded that lysine formed a chelate with the oxygen of the COO- and the N of the NH${}_{2}$. The observations that lysine interacts with the exchangeable cations suggested that lysine did not completely replace the interlayer Ca in the Lys-Mt complex, failing to restrict the interlayer expansion to block adsorption of pepsin. The lack of protein restriction of Lys-Mt could be due to a partial intercalation of lysine. The work in Yang et, al. [18] described the effect of lysine loading concentration and pH on the adsorption of lysine in montmorillonite. They proposed that adsorbed lysine interacted directly with the negatively charged sites on the montmorillonite surface. At low lysine concentrations (1 mM), the d(001) value was approximately 10.9 Å. Based on the lysine molecule dimension, the results indicated that lysine was only partially intercalated. The pH of the 1000 ppm lysine solution was 8.9; at this pH, lysine occurred as a cation [18], when interacting with the Ca-Mt dispersion. Low lysine interlayer loading and partial intercalation were also attributed to the rapid collapse of Lys-Mt at 100 ${}^{\circ }$C.

The thermogravimetric analysis of amino acids-montmorillonite complexes by Han et al. [19] showed a significant weight loss between 150 and 500 ${}^{\circ }$C for the lysine-montmorillonite, which was attributed to the amino acid decomposition. The interlayer stability of the Arg-Mt and Hist-Mt complexes showed a gradual decrease until 150 ${}^{\circ }$C; at this temperature, most of the interlayer water was removed, and the collapsed basal spacing at 300 ${}^{\circ }$C was due to the decomposition of the amino acids. The Chol-Mt and VB1-MT complexes maintained a more expanded interlayer spacing of 13–14 Å at 150 ${}^{\circ }$C than the amino acids-montmorillonite complexes, which indicated less interlayer water and more loading of choline and vitamin B1.

### 3.2. Improved AfB1 Adsorption by Nutrient-Montmorillonite in the Presence of Pepsin

The nutrient-Mt increased the amount of aflatoxin adsorbed in the interlayer in the presence of pepsin as a result of the nutrient-Mt complexes restricting the intercalation of pepsin. The efficiency varied possible due to the different nutrients’ loadings. Heating the Pep-nutrient-Mt complexes collapsed the interlayer to 13 Å in comparison to Pep-Ca-Mt, which maintained the interlayer at 15 Å. The significant difference in the d-values indicated that less pepsin was adsorbed in the nutrient-montmorillonite complexes than Ca-Mt.

At room temperature, Chol-Mt had similar d-value as AfB-Pep-Chol-Mt. The lack of expansion indicated that choline in the interlayer favored the adsorption of aflatoxin on the hydrophobic sites, but in the pepsin solution alone, the interlayer expanded to about 15.5 Å.

Among the five nutrient-Mt complexes, VB1-Mt was the most effective at improving the aflatoxin adsorption capacity and the affinity in the presence of the protein. The increased adsorption capacity from 0.35 mol/kg in Ca-Mt to 0.56 mol/kg in VB1-Mt in the presence of pepsin suggested that the nutrient-Mt potentially increased the adsorption of aflatoxin in animal experiments. Further in vivo trials are required to test the improved aflatoxin adsorption. The significant improvement of the Qmax and the affinity resembled previously observed isotherm values in simplified solution (water) [8]. Ca-Mt showed an L shaped-type isotherm in water with a Qmax of 0.41 mol/kg and Kd 1.4 μM${}^{-1}$. The VB1-Mt complex showed similar adsorption values, but the isotherm showed an S-type shape rather than the previously observed L-shaped isotherm.

### 3.3. Restriction of Interlayer Adsorption of Pepsin

A complete pepsin restriction to the interlayer by the nutrient-Mt was not achieved in this study. The d001 basal spacing of the nutrient-Mt complexes collapsed to 10 Å after heating at 300 ${}^{\circ }$C, except for vitamin B1, but all Pep-nutrient-Mt complexes remained at 13Å. When pepsin was the only organic compound in the solution, the FTIR data indicated the adsorption of the protein, but when aflatoxin and pepsin were present in the solution, adsorption of aflatoxin was favored. The nutrient-Mt complexes adsorbed less pepsin than the unmodified Ca-Mt as indicated by the XRD data.

When modified with lysine, the basal space of montmorillonite showed only a slight expansion in the presence of aflatoxin and pepsin. This increase could be a change in the arrangement or conformation of the lysine or room humidity effect.

VB1-Mt showed a higher d-value when both pepsin and aflatoxin were present in the solution. The high adsorption maxima, along with the reduced FTIR pepsin bands and the expanded basal spacing indicated a different aflatoxin molecule arrangement in the interlayer from the nearly flat monolayer observed in the AfB1-smectites formed in aqueous solutions. The adsorption isotherm was not L-shaped, but it resembled more an initial S-type shape.

### 3.4. Adsorption Mechanism

Deng et al. [4] demonstrated that the exchangeable cation strongly influenced the adsorption of aflatoxin. Based on the response of the C=O band to the type of exchange cations, it was demonstrated that there were two adsorption mechanisms between the aflatoxin molecules and the exchangeable cation: (1) an ion–dipole interaction at 0% humidity and (2) a hydrogen bonding between the hydration shell of the cation and the carbonyl groups. Small shifts were observed on monovalent cations (Na, K) exchanged montmorillonite near 1748 cm${}^{-1}$. In the FTIR of AfB1-Ca-Mt, the C=O band was observed at 1727 cm${}^{-1}$, which was a very similar band position observed for the nutrient-Mt complexes, suggesting that the adsorbed aflatoxin had a weak interaction with the interlayer organic nutrients. In contrast, the carbonyl band of aflatoxin adsorbed in the vitamin B1-montmorillonite remained at a similar position as the free aflatoxin at 1748 cm${}^{-1}$ [20], which indicated that an ion–dipole interaction was not the main adsorption mechanism for aflatoxins in the nutrient-Mt complexes. Based on the similar band positions of VB1-Mt compared to the free aflatoxin [4], the adsorption of aflatoxin was an interaction with the hydrophobic site in the montmorillonite, while vitamin B1 only served to maintain an optimal interlayer environment.

The band at 1235 cm${}^{-1}$ was observed only in the AfB1-VB1-montmorillonite complex, while a band at 1246 cm${}^{-1}$ was observed for the other AfB1-nutrient-Mt. Deng et al. [4] reported the presence of this band in all the different cation-montmorillonites, and the free aflatoxin had a group of bands from 1230–1227 cm${}^{-1}$, which were attributed to diverse in-plane and out-of-plane deformations of the CH.

## 4. Conclusions

Intercalating the five cationic and polar organic nutrients (arginine, histidine, lysine, choline, and vitamin B1) into the interlayer space of montmorillonite improved the AfB1 adsorption in simulated gastric fluid by restricting the adsorption of pepsin. Yet, pepsin was not completely blocked. Among the five tested nutrients, vitamin B1 was the most effective at blocking the adsorption of pepsin and increasing the adsorption capacity and affinity of aflatoxin in the simulated gastric solution. AfB1 molecules could access the interlayer spaceof the nutrient-Mt complexes. The nutrient had small effects on the bonding mechanism of aflatoxin molecules to montmorillonite in the complexes.

## 5. Materials and Methods

### 5.1. Montmorillonite, Nutrients, and Pepsin

The <2 μm clay fraction of a Ca-bentonite from Gonzales, Gonzales, TX, USA (labeled as 4TX) was used in this study. Montmorillonite was the dominant clay mineral. The montmorillonite (Ca-Mt) sample demonstrated a high aflatoxin binding capacity Qmax of 0.4 mol/kg in water [6].

Five commonly used nutrients in feed were selected as modifying organic anchors in the montmorillonite’s interlayer: three amino acids (arginine, histidine, and lysine), one vitamin (thiamine), and a vitamin-like essential nutrient (choline). These small nutrient molecules are either cationic or can be protonated, and they are suitable to occupy the negatively charged sites in smectite without competing with the neutral non ionic aflatoxin molecules. Thiamin chloride (vitamin B1), L-lysine, and choline chloride were purchased from Sigma Aldrich (Saint Louis, MI, USA). The L-arginine monohydrochloride and L-histidine monohydrochloride monohydrates were purchased from VWR. A stock solution of 1000 mg/mL was prepared in DI water for each organic compound, and the pH of each stock solution was recorded.

Pepsin (from porcine gastric mucosa) was obtained from Sigma Aldrich. The pepsin solution was prepared by dispersing 624 mg of pepsin in 500 mL of DI water [8]. The pepsin stock solution was centrifuged at 4000 rpm for 40 min to remove undissolved protein and to obtain a clear solution.

### 5.2. Synthesis of Nutrient-Montmorillonite Complexes

Ca-montmorillonite (Ca-Mt) dispersions, containing 10 mg of the clay mineral, were mixed with 10 mL of the 1000 mg/mL stock solution of each organic nutrient. The samples were shaken overnight on an orbital shaker. The nutrient-montmorillonite (nutrient-Mt) complexes’ dispersions were centrifuged at 4000 rpm for 40 min and washed twice with DI water. After removing the supernatant, 5 mL of DI water were added to each sample to obtain a 2 mg/mL clay dispersion (Table 2).

To examine if the nutrients intercalated in the montmorillonite, the basal spaces d(001) of the nutrient-montmorillonite complexes were monitored by XRD at elevated temperatures. The nutrient-Mt complex dispersions were air died on silicon wafers and placed in an Anton Paar reactor chamber XRK900 on a Bruker D8 advance diffractometer. The initial temperature in the chamber was set to 30 ${}^{\circ }$C, then the samples were heated from 50 to 200 ${}^{\circ }$C at 25 ${}^{\circ }$C intervals and from 200 to 300 ${}^{\circ }$C at 50 ${}^{\circ }$C intervals. XRD patterns were recorded at the predetermined temperature.

### 5.3. Loading Nutrient-Montmorillonite with AfB1 and Pepsin

The nutrient-Mt complexes were loaded with AfB1, pepsin, and AfB1 plus pepsin to check the interlayer accessibility. To prepare the AfB1-nutrient-Mt complexes, an aliquot of 0.5 mL of the nutrient-Mt dispersion, containing 1 mg of the complex, was mixed with 20 mL of an 8 ppm AfB${}_{1}$ solution prepared in DI water. The mixtures were shaken overnight, centrifuged at 4000 rpm for 30 min, and washed two times with DI water. A similar procedure was followed to prepare the pepsin loaded nutrient-Mt (Pep-nutrient-Mt) complexes and the complexes with both aflatoxin and pepsin (AfB-Pep-nutrient-Mt), using the same pepsin solution as in the isotherms (624 mg pepsin/500 mL).

All nutrient-montmorillonite complexes loaded with AfB1, pepsin, or AfB1+pepsin were air dried on zero background silicon slides for X-ray diffraction analysis (XRD) at room temperature. Then, the slides were heated at 300 ${}^{\circ }$C in a furnace for 1 h, cooled at room T, and the XRD patterns recorded again.

To investigate the interactions among the aflatoxin, nutrients, and montmorillonite, the montmorillonite complexes loaded with nutrients, aflatoxin, and the nutrient solutions were air dried on ZnS windows, and FTIR spectra were recorded in transmission mode in a Spectrum 100 Perkin Elmer FTIR spectrometer with 32 scans and a resolution of 2 cm${}^{-1}$ for each spectrum. The complexes were recorded at room and low humidity conditions. The chamber was purged with N${}_{2}$ to reduce moisture. The nutrient-Mt with AfB${}_{1}$ spectra were also recorded under 100% humidity by placing a piece of wet Kimwipe tissue between two ZnS discs.

### 5.4. Aflatoxin Adsorption Isotherms on Nutrient-Montmorillonite Complexes in the Presence of Pepsin

Aflatoxin adsorption isotherms in the pepsin solution of the nutrient-Mt complexes were used to evaluate their capacity in blocking proteins from the interlayer. The isotherms were prepared following the procedure described in Barrientos et al. [16]. Fifty microliters of nutrient-Mt complex dispersion (concentration of 2 mg/mL) were added for each isotherm point. An 8 mg/mL AfB1 solution in pepsin was prepared from an AfB1 stock solution (1000 mg/mL in acetonitrile). The 8 mg/mL AfB1 in pepsin solution was diluted with pepsin at each isotherm point (0.4, 1.6, 3.2, 4.8, 6.4, and 8.0 mg/mL) to a total volume of 5 mL. The aflatoxin concentration in the supernatant was analyzed using a Beckman Coulter DU800 UV-spectrophotometer at 365 nm. The adsorption data were fitted to the modified Langmuir (QKLM) model [4].

## Figures and Tables

**Table 1:** AfB1 adsorption isotherm fit parameters of nutrient-montmorillonite complexes in pepsin solution.

Nutrient-Mt Complex	Aflatoxin Adsorption			
	Q${}_{\max}$ (mol/kg)	K (μ M${}^{-1}$)	*b*	η ${}^{2}$
VB1-Mt	0.56	0.201	−0.086	0.91
Arg-Mt	0.42	0.045	−1.04	0.92
Chol-Mt	0.41	0.313	1.24	0.92
Hist-Mt	0.36	0.29	−0.121	0.83
Lys-Mt	0.48	0.063	−0.54	0.95
Ca-Mt *	0.32	0.065	−0.89	0.97

* Data from Barrientos-Velazquez et al. [16].

**Table 2:** Organic nutrients selected to modify the interlayer of a montmorillonite.

Name	Structure	Info	pH in Water
Choline	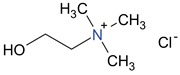	pKa: 13.9	6.73
Vitamin B1	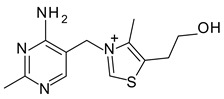	pKa: 4.8	3.67
Arginine	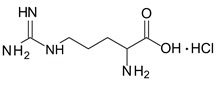	pKa${}_{1}$:2.17
		pKa${}_{2}$: 9.04	6.59
Histidine	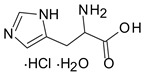	pKa${}_{1}$: 1.82
		pKa${}_{2}$: 9.04	4.41
Lysine	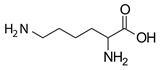	pKa${}_{1}$: 2.18
		pKa${}_{2}$: 8.95	8.09
